# Non-invasive cardiac stress studies may not offer significant benefit in pre-kidney transplant evaluation: A retrospective cohort study

**DOI:** 10.1371/journal.pone.0240912

**Published:** 2020-10-28

**Authors:** Durga Anil K. Kanigicherla, Talvinder Bhogal, Katie Stocking, Rajkumar Chinnadurai, Simon Gray, Saad Javed, Christien Fortune, Titus Augustine, Philip A. Kalra

**Affiliations:** 1 Manchester Institute of Nephrology & Transplantation, Manchester, United Kingdom; 2 Centre for Biostatistics, Faculty of Biology, Medicine and Health, The University of Manchester, Manchester, United Kingdom; 3 Department of Renal Medicine, Salford Royal NHS Foundation Trust, Hope Hospital, Salford, United Kingdom; 4 Division of Diabetes, Endocrinology and Gastroenterology, Faculty of Biology Medicine and Health, Manchester Academic Health Science Centre, University of Manchester, Manchester, United Kingdom; University Medical Center Utrecht, NETHERLANDS

## Abstract

**Background:**

Screening with cardiac non-invasive stress studies (NISS) prior to listing for kidney transplantation can help in identifying treatable coronary disease and is considered an integral part of pre-kidney transplant evaluation. However, few studies assessed their effectiveness in all patients evaluated for transplantation in clinical practice. To evaluate the role of NISS in pre-kidney transplant evaluation we analyzed their impact prior to waitlisting in 1053 adult CKD-5 patients consecutively evaluated in Greater Manchester, UK during a 6-year period.

**Methods:**

918 waitlisted patients were grouped based on presence or absence of Diabetes or Cardio-Vascular Disease (CVD): Group-1 (255 DM-/CVD-/NISS-), Group-2 (368 DM-/CVD-/NISS+) and Group-3 (295 with DM or CVD).

**Results:**

Group-2 patients had longer ‘time-to-listing’ (5.5months in Group-1 vs 6.9months in ‘Normal-NISS’ vs 9.9months in ‘Abnormal-NISS’, p<0.01) but none with ‘Abnormal-NISS’ needed coronary revascularization before listing. NISS was followed by revascularization in 8 Group-3 patients (3%). In multi-variate analyses, there was no association of NISS on death or MACE in listed patients. During follow up, Transplantation was the most significant factor associated with improved outcomes in all subgroups (HR:0.97, p<0.001). 135 patients were considered unsuitable for waitlisting, with NISS influencing management in 11 of these patients (8%).

**Conclusions:**

Pre-kidney transplant evaluation with NISS influenced clinical management in 19 of 1053 (2%) patients. Screening with NISS added limited benefit but contributes to significant delays in listing and adding resource implications. Further studies are needed to assess clinical and cost effectiveness of NISS in pretransplant evaluation to optimize outcomes and resources.

## Background

Coronary artery disease is the leading cause of death in patients with end-stage renal disease (ESRD). Renal transplantation reduces and halts cardiovascular disease progression and mortality in patients with ESRD [1]. Risk of a major cardiac event rises in the early post-transplant period and declines to a lower rate subsequently [2]. Screening for ischemic heart disease with Non-Invasive Stress Studies (NISS) is considered useful in identifying those who could benefit from revascularization prior to transplantation. This could help in preventing cardiovascular events immediately post-transplantation as well as in the short to medium term. Some current guidelines [3–6] recommend screening for ischemic heart disease in asymptomatic transplant candidates prior to listing for kidney transplantation whilst some suggest that it may be helpful to use aggregate coronary artery disease (CAD) risk factors to target screening in patients with the highest pre-test likelihood of significant CAD. Many centers screen for occult ischemic heart disease in candidates prior to listing for kidney transplantation. However, previous studies investigating occult coronary disease noted mixed results, and guidelines are derived mostly from observational studies.

Screening with NISS using risk stratification is proposed to potentially avoid unnecessary invasive investigations in potential recipients with low risk. Studies evaluating the clinical application of screening tests based on risk stratification noted that a relatively low proportion of screened patients underwent pre-transplant intervention [7–10]. In these studies, between 3 and 9% of apparent ‘high risk’ transplant candidates underwent coronary angioplasty or bypass grafting prior to listing. Pooled estimates of sensitivity were 0.69 for myocardial perfusion studies and 0.80 for Dobutamine stress echocardiography, with respective specificities of 0.77 and 0.89 [11]. Local availability of screening tests and the expertise in interpreting them are considered important when deciding upon the suitability of a screening program for myocardial ischemia [12]. Some observational studies showed significant positive association between NISS and subsequent cardiac events [13], whereas many others have not [14–16]. Also, in some studies the presence and severity of coronary disease on angiography was not predictive of survival [17]. There are very few randomized studies that have evaluated the benefit of coronary revascularization over standard medical therapy [18]. A recent study by Goyal et al. [19] from the National Inpatient Sample in the US showed that rates of perioperative MACE after kidney transplantation remain low on an absolute level and the data suggest that post- operative MACE may be driven more by heart failure than acute coronary syndromes. Therefore, clinicians wrestle with the question of whether screening for occult ischemic heart disease is effective in preventing subsequent vascular events.

Current guidance is drawn from data that included patients with preexisting cardiovascular disease or who were diabetic in the same cohorts. Factors considered to be of ‘high-risk’ varied between various studies [20] and in various guidelines. It is challenging to draw conclusions as to what factors constitute ‘high-risk’ in patients without symptoms or diabetes or pre-existing cardiovascular disease. It is also unclear if screening with NISS poses additional challenges with postponement of transplantation or if patients are subjected to additional investigations that may or may not lead to therapeutic benefits following the non-invasive studies. There is paucity of evidence related to time taken to waitlist patients and if these screening investigations add additional time to this process.

We aimed to evaluate the role of non-invasive stress studies in pre-kidney transplant evaluation for patients categorized by risk–primarily based on the presence or absence of three factors a) diabetes, b) symptoms of cardiovascular disease and c) pre-existing history of vascular disease. We assessed the time taken to activate patients on the list, additional investigations needed prior to listing and the association of subsequent cardiac events and mortality with baseline factors including NISS results. For the purpose of clarity, we report our findings in 2 distinct groups based on features at the time of initial assessment: 1) Patients without DM or CVD (Groups 1 & 2) and those with either pre-existing DM or CVD (Group 3). To assess the overall benefits of NISS in all patients evaluated for kidney transplantation, we describe the ‘Unlisted’ patients to help draw comparisons and conclusions.

## Methods

The region of Greater Manchester has a population of about 3 million and patients with advanced CKD receive renal services through one of the 2 centers: Manchester University NHS Foundation Trust and Salford Royal NHS Foundation Trust. Patients who are deemed suitable for transplantation are referred to the Transplantation centre at Manchester Royal Infirmary and entered on to the national list for kidney transplantation. Patients considered to be of very high risk or with factors that impose challenges to kidney transplantation are not listed after multidisciplinary reviews.

Clinical Data was collected during a clinical evaluation project in the Transplant Recipient Evaluation Cohort for patients attending a dedicated ‘Nephrology-Transplant Recipient Evaluation Clinic’ between 2009 and 2014. Data was censored at the end of 2016. Deidentified data from the project was used for this study. The study and publication of results was approved by the Research and Innovation Board at Manchester University NHS Trust.

### Patient population

All patients underwent standard pretransplant screening based on phenotypic characteristics (S1 Fig). We included all adult patients aged ≥18 years who were consecutively referred for listing for kidney transplantation.

Most asymptomatic patients aged 50 years or more, those with diabetes mellitus and or with pre-existing cardiovascular disease underwent non-invasive stress studies according to local protocol. Relative contraindications for transplantation included presence of untreatable reversible ischemia, severe reduction in left ventricular ejection fraction (<30%), untreatable severe peripheral vascular disease, psychosocial factors, active infection and recent malignancy. Consequently, patients with these co-morbidities appear in the ‘Unlisted’ cohort. Waitlisted patients were grouped into the following cohorts to help analyze the role of NISS and outcomes:

- Group 1: No DM or CVD and No NISS–(n = 255)
- Group 2: No DM or No CVD and NISS+ (n = 368)
- Group 3a: DM+CVD- (n = 139)
- Group 3b: CVD+ (±DM) (n = 156)

Patients who had features of ischemia on NISS were evaluated by cardiologists and underwent coronary angiography if the ischemic burden in the stress study was noted to be significant. If there was evidence of significant occlusive coronary disease, revascularization with percutaneous intervention (PCI) or coronary artery bypass grafting (CABG) was undertaken. The patient pathway during pretransplant evaluation and prior to decision making is presented in S1 Fig. In terms of myocardium at risk, reversible ischemia in 3 or more segments was considered as a threshold for coronary angiography. If eGFR at the time of assessment was very low (10ml/min/1.73m^2^ or less) coronary angiography was undertaken on a case by case basis if symptoms of angina were significant or if features of ischemia on stress study were severe. In asymptomatic patients with only moderate ischemia a second form of NISS was used at the discretion of the cardiologist to validate the abnormalities noted on the first NISS prior to proceeding with coronary angiography.

In this study, both deceased and living donor kidney transplant recipients were included, but patients being considered for simultaneous kidney and pancreas transplants were excluded. Most patients who underwent transplantation received ‘standard induction’ with interleukin-2 receptor antagonist (Basiliximab) and dual maintenance immunosuppression with a calcineurin inhibitor, Tacrolimus and Mycophenolate Mofetil. A bolus of Methylprednisolone was given intra-operatively followed by oral prednisolone for 5 days. Information was obtained from the prospectively recorded dataset maintained at the Manchester Transplant Centre. All information recorded at the time of evaluation was part of routine practice to determine fitness for transplantation. Baseline variables included demographics, primary disease, Charlson comorbidity index [21], AHA risk factors [4] (3 or more of the 5 factors including age, smoking, renal replacement therapy at time of evaluation, hypertension and hyperlipidemia), and biochemical parameters.

### Timepoints, events and endpoints

Attendance in N-TREC was used as reference point. Patients were followed up until death or transfer to another centre or until last census. The data of 12 patients who were transferred to another renal centre was included as at the time of most recent follow up. The main clinical outcome was the composite endpoint of ‘major Adverse Cardiac event’ (MACE) after listing and / or death. MACE included development of acute myocardial infarction, or angina needing coronary revascularization, and death constituted all-cause mortality.

### Statistical analyses

Continuous data was described using means and standard deviations (SD) or median and inter-quartile range (IQR). For categorical variables, proportions and frequencies were reported. Where appropriate, comparisons between groups were made by using Two-Sample T-Tests or Mann-Whitney U-Tests to compare continuous variables and Chi-Square or Fisher’s Exact Tests for categorical.

Comparative analysis was undertaken between different groups. Time to listing was calculated by computing the time between N-TREC and activation on the list. Comparisons of time to listing was made between patients in the different groups using the Kruskal Wallis Test. Pairwise comparisons were made using Dunns Test with a Bonferroni adjustment to account for multiple testing.

Survival analysis, with respect to the time to waitlisting and time to the combined clinical endpoint, was undertaken using Kaplan Meier curves and the Log-Rank test. Cox proportional hazards models were used to calculate the hazard ratio (HR) and 95% confidence interval (CI). In order to construct the final model for survival analyses, univariate Cox models including clinical variables, Charlson comorbidity index, pre-emptive evaluation, cardiovascular risk factors, biochemical variables, NISS and subsequent transplantation were used. Each covariate was added into a univariate model and statistically significant parameters demonstrating association with the composite end point were included in multivariate analysis. A p-value of <0.05 was considered to be statistically significant. All analyses were carried out using Stata 14.

## Results

1053 patients were referred to N-TREC for assessment and entry onto the kidney transplant waitlist. 135 patients did not proceed to waitlisting for various reasons. 918 patients were listed for kidney transplantation over the 6-year period and were followed for a mean of 50.4 months. The mean age was 49.9 years. Patients who were listed were entered on waitlist after a median of 7.2 months from the initial clinic consultation in N-TREC. 719 of these patients underwent evaluation during ‘preemptive’ stage of CKD. Of these 106 (15%) patients started dialysis for progressive stage 5 CKD during the evaluation period, before they were registered on the list.

**Fig 1:**
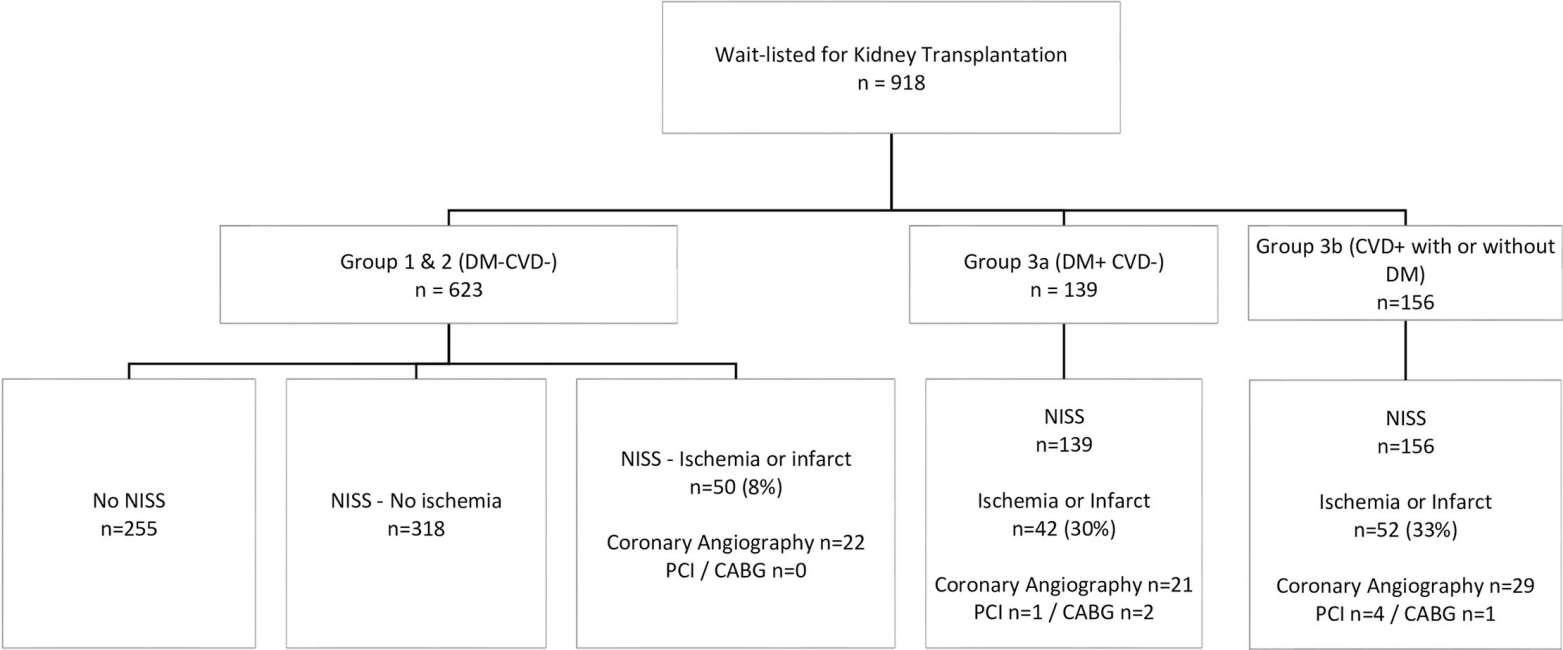
Waitlisted candidates: Non-invasive stress studies (NISS), additional cardiac investigations and coronary interventions.

The data for subgroups of patients with cardiac investigations, and the subsequent interventions undertaken are shown in Figs 1 and 5. NISS were undertaken in 791 patients (75%). These comprised of Technetium myocardial perfusion scan in 64%, Rubidium SPECT study in 20%, and Dobutamine stress echocardiography in 16% patients. 179 of these 791 (23%) patients were noted to have an abnormal stress study–with prior myocardial infarct in 29, combined infarct and stress-induced ischemia in 11, and stress-induced ischemia in 139 patients. Cardiologist advice was sought in all patients and 98 patients (55%) with signs of significant reversible myocardial ischemia on NISS underwent coronary angiography. Significant coronary artery disease influenced subsequent management in 19 patients (2% of the whole cohort)–revascularization in 14 patients (9 patients had PCI and 5 had CABG) and 5 other patients had multi-vessel coronary disease but could not proceed with revascularization because of comorbidities and were excluded from transplant listing.

537 patients (58%) received either a living donor or deceased donor kidney transplant up until last follow-up, with a mean delay of 26.8 months from the time of evaluation. In the waitlisted group 175 (19%) patients had a combined clinical end point during follow up– 87 (9%) patients with MACE and 116 (13%) patients died. 18 deaths followed transplantation (3% of 537 patients) and 98 occurred whilst on waitlist or after suspension on the list (26% of 381 patients). 80 out of 135 patients (59%) in the ‘Unlisted’ group developed a combined endpoint– 47 (35%) patients with MACE and 55 (41%) patients died.

### Waitlisted candidates without DM or CVD (Groups 1 and 2)

The baseline features of 623 patients without prior history of DM or CVD are shown in Table 1. Patients in Group 2 (who had NISS, n = 368) were older and had a higher Charlson comorbidity index, hypertension and smoking history. A higher proportion of patients who had NISS were receiving statins. 90% of patients aged 50 years or more underwent NISS. 50 of these 368 patients (14%) had some evidence of myocardial ischemia with or without infarction on NISS. 20 patients underwent coronary angiography. 21 other patients with reversible ischemia underwent a second form of NISS at the discretion of the cardiologist and 2 of these 21 patients needed coronary angiography. None of these 22 patients who underwent coronary angiography were deemed to have obstructive disease that needed angiographic intervention prior to waitlisting. Among baseline variables, the only significant difference was with male preponderance in patients with abnormal NISS. None of the other baseline variables were associated with abnormalities on NISS (S1 Table).

**Table 1:** Baseline characteristics in waitlisted candidates without diabetes or cardiovascular disease (Groups 1 & 2) according to NISS status.

		Stress Test		P
		No	Yes	
		Group 1	Group 2	
		(n = 255)	(n = 368)	
Age: years		36.7 (11.1)	54.1 (11.8)	< 0.001
BMI		26.0 (4.5)	26.8 (4.9)	0.04
Male (%)		152 (59.6)	191 (51.9)	0.06
Hypertension (%)		209 (82.0)	327 (88.9)	0.02
Non-Smoker (%)		189 (74.1)	210 (57.1)	< 0.001
Ethnicity (%)	Caucasian	197 (77.3)	293 (79.6)	0.48
Primary Disease (%)	Immune Kidney Disease	71 (27.8)	117 (31.8)	<0.01
	Hypertensive CKD	30 (11.8)	58 (15.8)	
	Polycystic Kidney Disease	33 (12.9)	67 (18.2)	
	Other	65 (47.5)	76 (34.3)	
Previous Transplant (%)		32 (12.6)	56 (15.2)	0.35
Pre-emptive evaluation (%)		211 (82.8)	279 (75.8)	0.38
On Dialysis at evaluation (%)		44 (17.2)	89 (24.2)	
Pulmonary Disease (%)		13 (5.1)	34 (9.2)	0.05
Peptic ulcer disease (%)		6 (2.4)	21 (5.7)	0.04
Liver Disease (%)		10 (3.9)	12 (3.3)	0.66
Previous Cancer (%)		8 (3.1)	27 (7.3)	0.03
HIV (%)		3 (1.2)	2 (0.5)	0.40
Charlson Comorbidity Index		2 (2,2)	2 (2, 3)	< 0.001
AHA risk factors (3 or more)		80 (31.4)	187 (50.8)	<0.001
RAS inhibitors (%)		168 (65.9)	210 (57.1)	0.03
Statin (%)		97 (38.0)	203 (55.2)	< 0.001
eGFR (ml/min/1.73m^2^)		12.3 (5.0)	11.6 (4.6)	0.08
Calcium (mmol/L)		2.3 (0.2)	2.3 (0.2)	0.36
Phosphate(mmol/L)		1.4 (1.2, 1.7)	1.4 (1.2, 1.7)	0.38
PTH (ng/L)		199 (116, 343)	200 (118, 355)	0.80
Albumin (g/L)		40.4 (5.9)	39.7 (5.6)	0.12
CRP (mg/L)		3.2 (1.4, 6)	5 (2.5, 8.2)	< 0.001
Total Cholesterol (mmol/L)		4.8 (4.0, 5.6)	4.6 (2.4, 11.6)	0.17
HDL Cholesterol (mmol/L)		1.2 (1.0, 1.5)	1.3 (1.1, 1.7)	0.03
Total / HDL Cholesterol (mmol/L)		3.8 (3.0, 4.7)	3.4 (2.8, 4.4)	<0.01

Results shown as Mean (Standard Deviation), Median (IQR) for continuous variables and Number (Frequency) for categorical variables.

p-Value from Two Sample T-Test, Mann-Whitney U Test and Chi-Squared or Fisher’s Exact test as appropriate.

**Fig 2:**
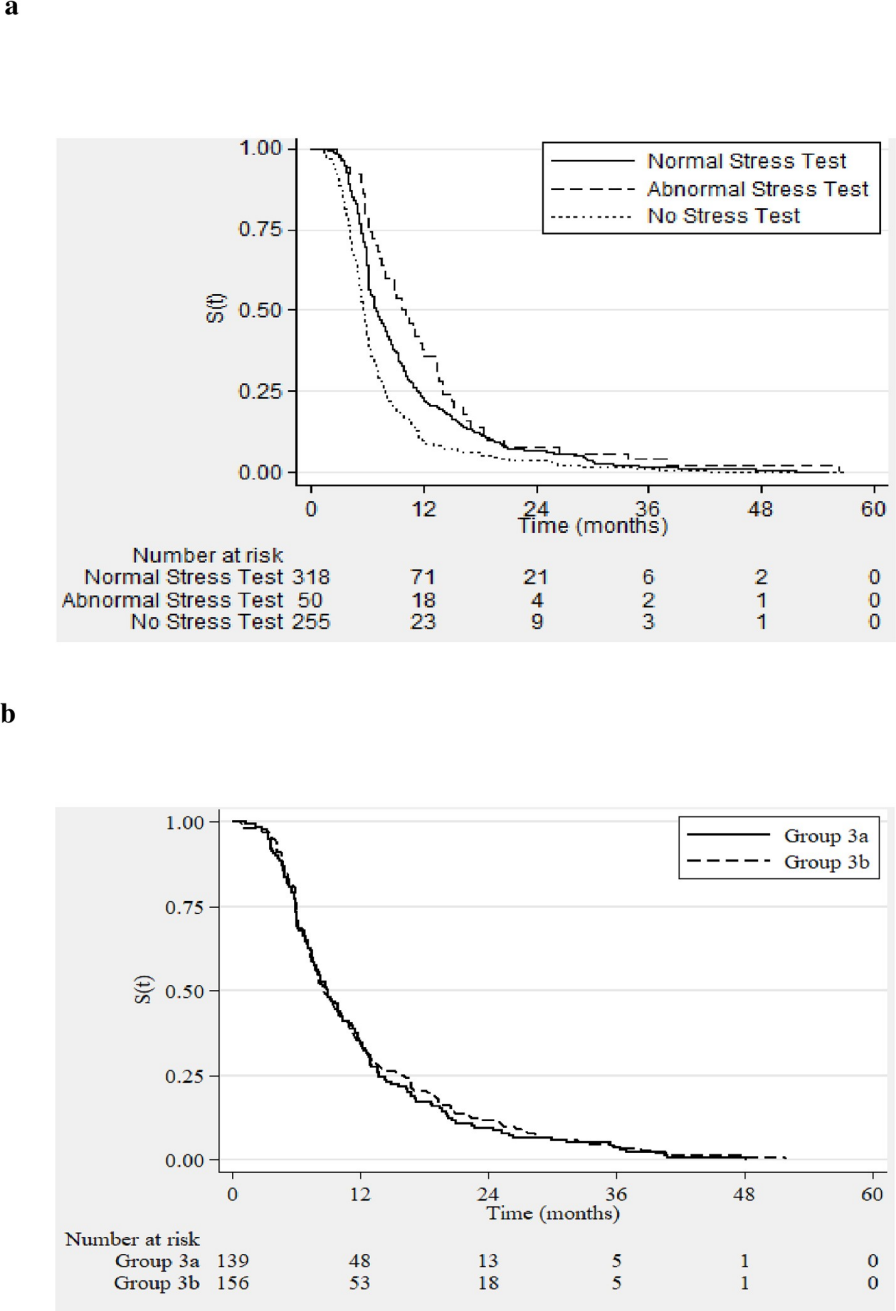
**A.** Time taken for transplant waitlisting in waitlisted candidates–Group 1 (No stress test), Group 2 with normal stress test & Group 2 with abnormal stress test; Time 0 –transplant recipient evaluation clinic. **B**. Time taken for transplant waitlisting in waitlisted candidates–Group 3a (DM+CVD-) and Group 3b (CVD+); Time 0 –transplant recipient evaluation clinic.

Fig 2A illustrates the time taken for listing in these groups. The median time taken for listing patients who had a normal NISS was 6.9 months (IQR: 5.3, 11.4). In comparison, the median time taken to waitlist in patients who had an abnormal NISS was 9.9 months (IQR: 6.3, 14.0). For patients who did not have the stress study the median time taken for listing was 5.5 months (IQR: 4.1, 7.9). A significant difference was found between the median follow up time in the 3 groups (p<0.001). Dunn’s test was used to conduct non-parametric pairwise comparisons of the independent groups (with a Bonferroni Adjustment in order to account for multiple testing). A significant difference was found between the median times in ‘abnormal’ vs ‘normal NISS’ groups (p = 0.02), ‘normal’ vs ‘no NISS’ groups (p<0.01) and ‘abnormal’ vs ‘no NISS’ groups (p<0.001).

**Fig 3:**
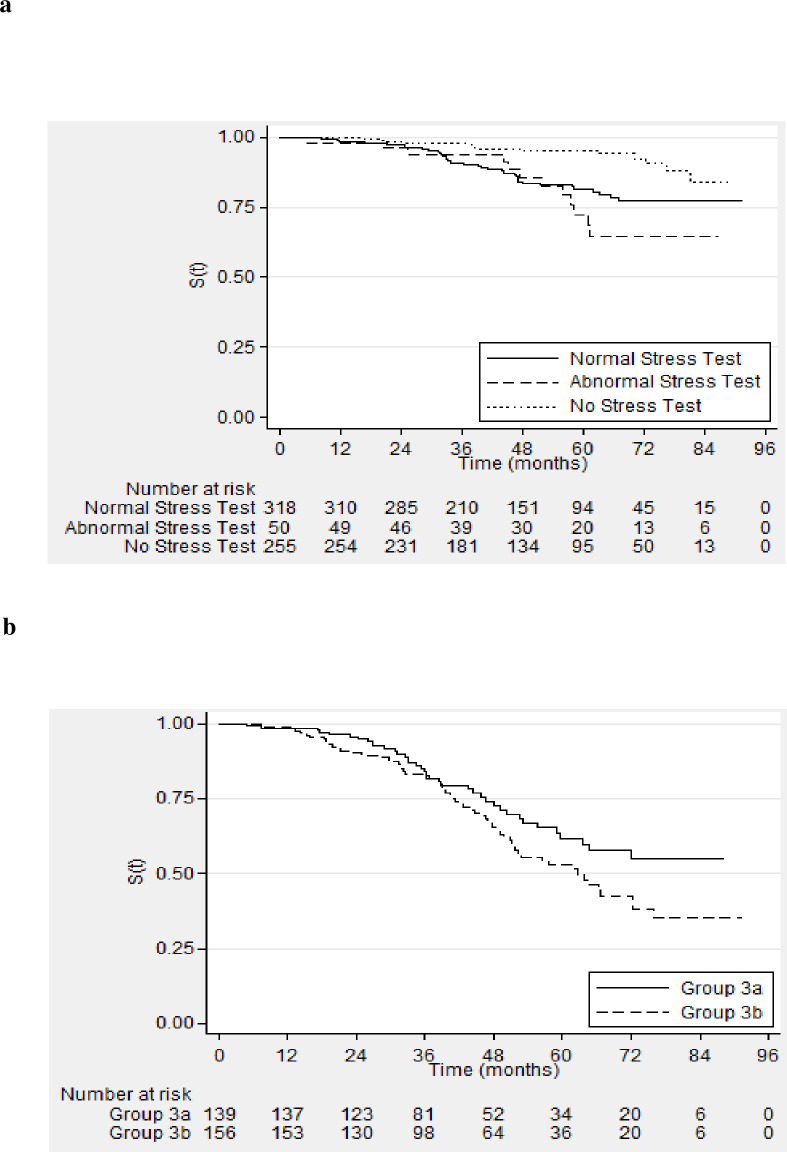
**A.** Survival free of composite endpoint in waitlisted candidates–Group 1, Group 2 with normal stress test & Group 2 with abnormal stress test; Time 0 –transplant recipient evaluation clinic. **B**. Survival free of composite endpoint in waitlisted candidates–Group 3a (DM+CVD-) and Group 3b (CVD+); Time 0 –transplant recipient evaluation clinic.

**Fig 4:**
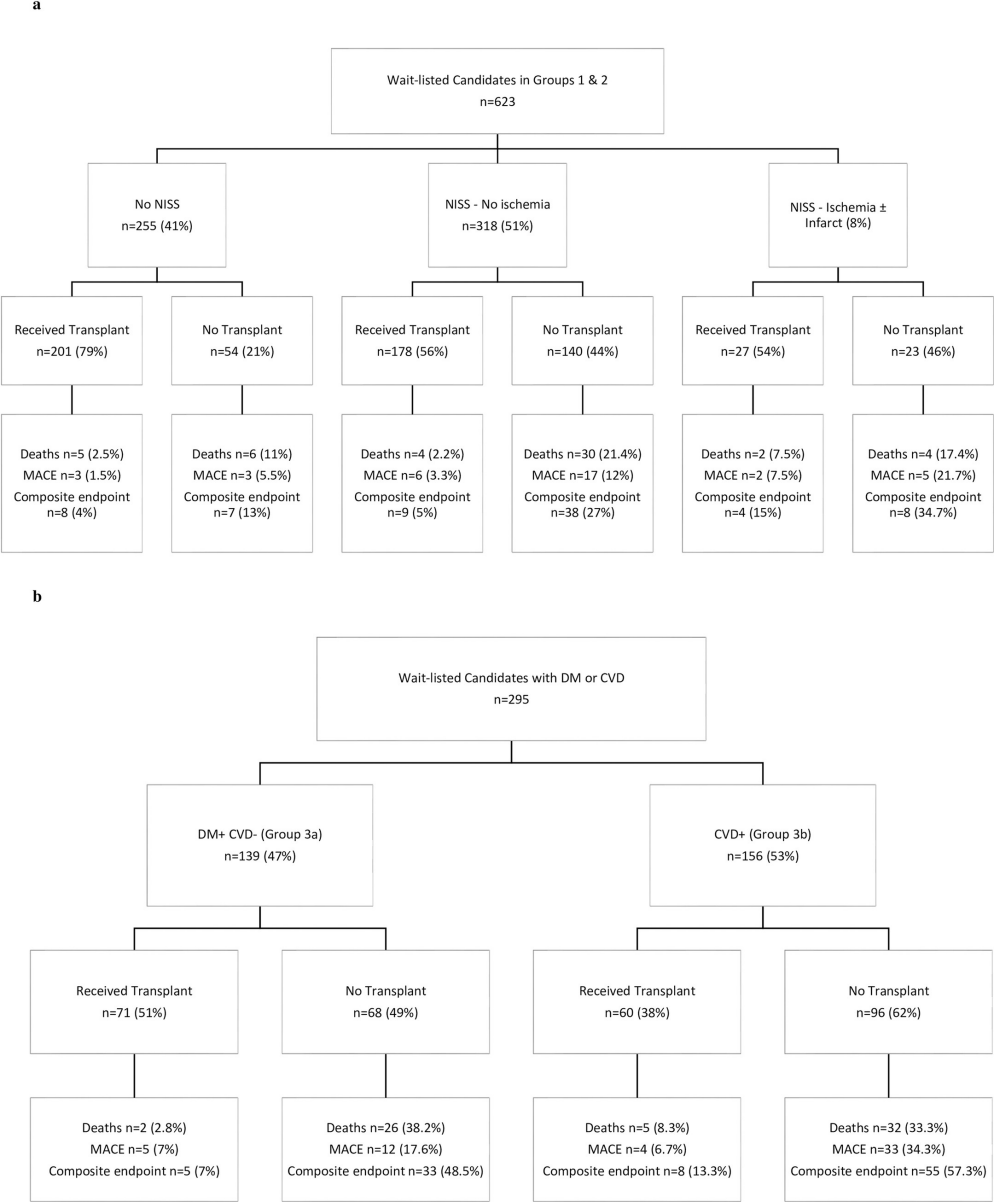
**A.** Clinical outcomes in waitlisted candidates–Group 1 & 2 based on non-invasive stress study. **B**. Clinical outcomes in waitlisted candidates–Group 3a (DM+ CVD-) and Group 3b (CVD+).

Of these 623 patients, 74 (12%) had a composite end point during follow up–MACE and/or death (Fig 3A). Crude event rates were 1%, 7% and 24% at 1, 3 and 5-year follow up period. Log rank analyses showed significant difference in the event-free rate between the patients who had a normal stress test, an abnormal stress test and those that did not receive a stress test (p<0.001). However, there was no significant difference in the log-rank test in event-free rate between patients who had normal stress test and abnormal stress test (p = 0.30). The time from transplantation (in months) was used as a time-dependent co-variate in survival analysis. The Charlson comorbidity index, AHA risk factors (3 or more) and time after subsequent transplantation were significantly associated with event-free survival (Table 2). Overall clinical outcomes during follow up are shown in Fig 4A.

**Table 2:** Cox-proportional hazard analysis for risk of composite endpoint in waitlisted candidates in Groups 1 & 2, multi-variate analysis (including factors considered relevant / significant in univariate analyses).

	HR (95% CI)	p
No Stress Test	0.68 (0.37, 1.25)	0.22
Charlson Co-morbidity Index	1.43 (1.13, 1.80)	<0.01
AHA Risk Factors (3 or more)	1.97 (1.19, 3.26)	0.01
Subsequent Transplantation	0.97 (0.96, 0.98)	<0.001

* Transplantation treated as a time dependent covariate (in months).

### Waitlisted candidates with prior diabetes (Group 3a) or cardiovascular disease (Group 3b)

Baseline characteristics of 139 patients with diabetes mellitus and 156 patients with prior cardiovascular disease, are shown in Table 3. Cardiovascular disease comprised pre-existing ischemic heart disease in 50%, peripheral vascular disease in over a third and cerebrovascular disease in just over a quarter of patients. 44% of patients in Group 3b had pre-existing Diabetes. Evaluation was undertaken in ‘pre-emptive’ stage of CKD-5 in 77% of these patients. As one would expect, risk factors including age, BMI, hypertension, Charlson comorbidity index, and smoking were more prevalent in these groups compared to patients in groups 1 and 2. Interestingly, there were only few baseline differences (gender and non-Caucasians) between those with DM or CVD.

**Table 3:** Baseline characteristics in waitlisted candidates with diabetes or pre-existing cardiovascular disease.

		Diabetes Mellitus	Pre-existing Cardiovascular disease	p
		Group 3a	Group 3b	
		(n = 139)	(n = 156)	
Age		53.4 (13.3)	58.2 (11.6)	<0.01
BMI		28.8 (4.5)	28.0 (4.8)	0.18
Male (%)		84 (60.4)	114 (73.1)	0.02
Hypertension (%)		128 (92.1)	146 (93.6)	0.62
Non-smoker (%)		89 (64.0%)	67 (43.0%)	<0.001
Caucasians (%)		98 (70.5)	128 (82.1)	0.02
Primary Disease (%)	Diabetes	99 (71.2%)	69 (44.2%)	
	Non-diabetic CKD	40 (28.8)	87 (55.8)	
Previous Transplant (%)		7 (5.0)	19 (12.2)	0.03
Pre-emptive evaluation (%)		109 (78.4)	120 (76.9)	0.76
On Dialysis at evaluation (%)		30 (21.6)	36 (23.1)	
Pulmonary Disease (%)		10 (7.2)	14 (8.9)	0.58
Peptic ulcer disease (%)		3 (2.2)	10 (6.4)	0.07
Liver Disease (%)		5 (3.6)	3 (3.8)	0.91
Previous Cancer (%)		7 (5.0%)	12 (7.7%)	0.36
CCF (%)		0	9 (5.8%)	<0.01
Charlson Comorbidity Index		4 (4, 4)	5 (3, 6)	0.06
PVD (%)		0	60 (38.5%)	<0.001
IHD (%)		0	78 (50.0%)	<0.001
CVD (%)		0	42 (26.9%)	<0.001
AHA risk factors (3 or more)		80 (57.6)	103 (66%)	0.13
RAS inhibitors (%)		93 (66.9)	117 (75.0)	0.13
Statin (%)		113 (81.3)	129 (82.7)	0.76
eGFR (ml/min/1.73m^2^)		13.3 (4.8)	12.6 (4.6)	0.22
Calcium (mmol/L)		2.2 (2.1, 2.3)	2.3 (2.2, 2.4)	0.09
Phosphate (mmol/L)		1.4 (1.2, 1.6)	1.4 (1.2, 1.7)	0.83
PTH (ng/L)		190 (121, 347)	208 (139, 357)	0.36
Albumin (g/L)		37.6 (6.4)	38.9 (5.4)	0.06
CRP (mg/L)		4 (2, 7)	4.8 (2–8)	0.73
Total Cholesterol (mmol/L)		4.1 (3.5, 5.0)	3.8 (3.1,4.9)	0.05
HDL Cholesterol (mmol/L)		1.2 (1.0, 1.6)	1.2 (0.9, 1.4)	0.13
Total / HDL Cholesterol (mmol/L)		3.5 (2.6, 4.7)	3.3 (2.7, 4.0)	0.39

Results shown as Mean (Standard Deviation), Median (IQR) for continuous variables and Number (Frequency) for categorical variables.

p-Value from Two Sample T-Test, Mann-Whitney U Test and Chi-Squared or Fisher’s Exact test as appropriate.

The median time taken for listing in Group 3a (n = 139) was 8.9 months (IQR: 5.9, 13.7). For patients in Group 3b (n = 156), it was 8.6 months (IQR: 6.0, 15.8) and there was no significant difference in ‘time to listing’ between the two groups (p = 0.78) (Fig 2B). 94 of the 295 (32%) patients were noted to have abnormal NISS (myocardial ischemia with or without an infarct). 47 of these patients proceeded to coronary angiography. 31 other patients underwent a 2^nd^ form of NISS to corroborate the ischemic burden noted on the first NISS. Only 3 of these patients then proceeded with coronary angiography. In total, of 50 of these 94 patients who underwent coronary angiography, 8 patients were found to have significant disease– 5 patients underwent intervention with angioplasty and stent insertion and 3 had coronary bypass grafting.

Of the 295 patients with diabetes or cardiovascular disease, 101 (34.2%) had a combined end point– 65 patients died (22%) and 54 (18%) patients had MACE (Fig 3B). Crude event rates were 2%, 17% and 52% at 1, 3 and 5-year follow up period. Log rank analysis showed a difference approaching significance with higher rates of adverse events in patients with cardiovascular disease (p = 0.055). Charlson comorbidity index, preexisting cardiovascular disease, AHA risk factors (3 or more) and transplantation were identified as important covariates in the univariate analysis. However, none of the factors other than transplantation (HR 0.95, 95% CI 0.94–0.96, p<0.001) were found to be statistically significant in a multivariate model (Table 4). Overall clinical outcomes during follow up are shown in Fig 4B.

**Table 4:** Cox-proportional hazard analysis for risk of composite endpoint of MACE or death in waitlisted candidates in Groups 3a and 3b: Multi-variate analysis (including factors considered relevant / significant in univariate analyses).

	HR (95% CI)	p
Charlson Index	1.22 (0.87, 1.32)	0.18
Group 3b	1.08 (0.72, 1.62)	0.72
AHA 3 or more risk factors	1.12 (0.86, 1.25)	0.84
Subsequent Transplantation*	0.95 (0.94, 0.96)	< 0.001

* Transplantation treated as a time dependent covariate (in months).

### Unlisted candidates

**Fig 5:**
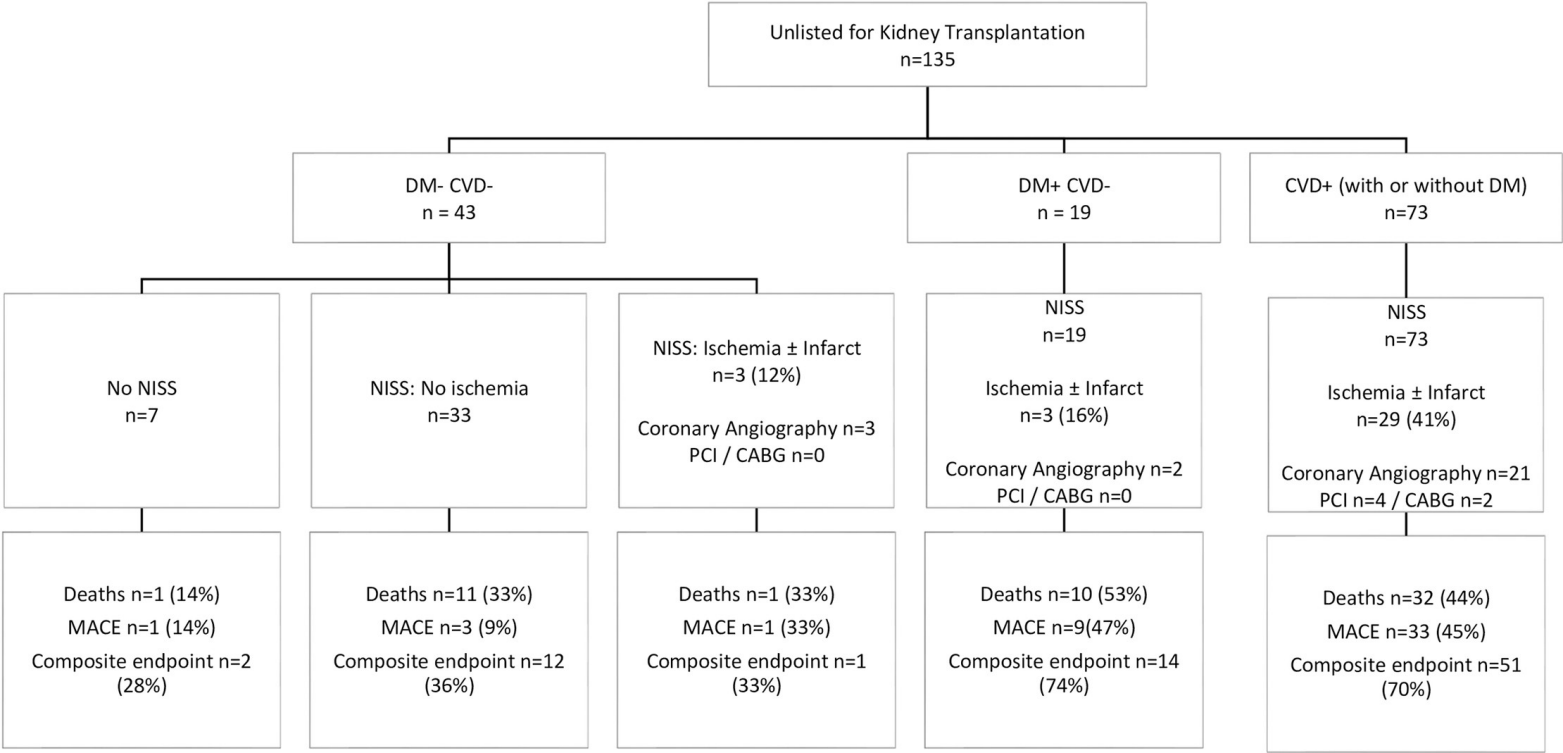
Unlisted candidates: Non-invasive stress studies, additional cardiac investigations, coronary interventions and outcomes.

135 of 1053 patients (13%) who underwent evaluation were not considered suitable for listing during the study period. Baseline characteristics are outlined in Table 5 and cardiac investigations, interventions and outcomes are shown in Fig 5. More than half of these patients had prior cardiovascular disease. Decision to not list patients was based on multiple factors. Patients who were not waitlisted had higher proportion of all the risk factors that could be associated with poorer outcomes–including higher age, higher Charlson comorbid indices, more with pre-existing DM or CVD, and less proportion of patients in the pre-dialysis phase of kidney disease. Only 7 of the 135 patients did not undergo NISS and NISS per se were followed by coronary interventions in 6 out of 126 patients in this group. As would be expected, outcomes were substantially poor in this group compared to those in waitlisted groups. 70% of those with preexisting DM or CVD developed a composite endpoint during follow up. Crude event rates were 16%, 50% and 80% at 1, 3 and 5-year follow up period.

**Table 5:** Baseline characteristics in unlisted candidates.

		Unlisted Candidates without DM or CVD	Diabetes Mellitus +	Cardiovascular disease +	p
		(n = 43)	(n = 19)	(n = 73)	
Age		57.3 (11.9)	60.9 (13.6)	58.2 (10.3)	0.19
BMI		27.2 (4.9)	29.6 (5.6)	29.1 (4.6)	0.16
Male (%)		25 (58)	12 (63)	42 (58)	0.69
Hypertension (%)		37 (86)	18 (95)	64 (87)	0.61
Non-smoker (%)		22 (51)	6 (32)	24 (33)	0.12
Caucasians (%)		33 (77)	13 (68)	50 (68)	0.61
Primary Disease (%)	Diabetes	0	19 (100)	41 (56)	
	Non-diabetic CKD	43 (100)	0 (0)	32 (44)	
Previous Transplant (%)		8 (19)	3 (16)	6 (8)	0.23
Pre-emptive evaluation (%)		26 (60)	14 (74)	42 (58)	0.43
On Dialysis at evaluation (%)		17 (40)	5 (26)	31 (42)	
Pulmonary Disease (%)		7 (16)	5 (26)	9 (12)	0.32
Peptic ulcer disease (%)		1 (2)	0 (0)	2 (3)	0.84
Liver Disease (%)		2 (4)	2 (11)	4 (6)	0.65
Previous Cancer (%)		9 (21)	4 (21)	5 (7)	0.05
CCF (%)		0 (0)	0 (0)	20 (27)	<0.001
Charlson Comorbidity Index		2 (2,4)	4 (4,5)	5 (4, 6)	<0.01
PVD (%)		0	0 (0)	29 (40)	<0.001
IHD (%)		0	0 (0)	31 (42)	<0.001
CVD (%)		0	0 (0)	29 (40)	<0.001
AHA risk factors (3 or more)		25 (58)	17 (89)	59 (81)	<0.01
RAS inhibitors (%)		15 (35)	6 (32)	26 (36)	0.52
Statin (%)		13 (30)	13 (68)	44 (60)	<0.01
eGFR (ml/min/1.73m^2^)		9.8 (3.2)	9.7 (2.2)	8.9 (4.1)	0.53
Calcium (mmol/L)		2.3 (2.2, 2.4)	2.2 (2.1, 2.4)	2.3 (2.1, 2.4)	0.39
Phosphate (mmol/L)		1.3 (1.0, 1.6)	1.3 (1.1, 1.6)	1.4 (1.2, 1.7)	0.50
PTH (ng/L)		189 (126, 418)	255 (135, 355)	201 (141, 362)	0.94
Albumin (g/L)		39 (34, 44)	38 (32, 43)	34 (31, 40)	0.01
CRP (mg/L)		6 (4, 9)	5 (4, 8)	8 (5, 11)	0.01
Total Cholesterol (mmol/L)		4.5 (3.8, 5.1)	4.3 (3.2, 5.3)	4.2 (3.4, 5.1)	0.42
HDL Cholesterol (mmol/L)		1.4 (1.2, 1.6)	1.1 (1.0, 1.5)	1.1 (1.0, 1.5)	0.06
Total / HDL Cholesterol (mmol/L)		3.2 (3.1, 3.3)	3.6 (3.3, 3.9)	3.4 (3.3, 3.6)	0.11

Results shown as Mean (Standard Deviation), Median (IQR) for continuous variables and Number (Frequency) for categorical variables.

p-Value from Two Sample T-Test, Mann-Whitney U Test and Chi-Squared or Fisher’s Exact test as appropriate.

## Discussion

Screening for coronary artery disease is considered an essential part of evaluation for patients with advanced kidney disease prior to kidney transplantation. However, practice varies widely across centers [18], partly because of the variable quality of evidence that belies the recommendations. Screening could be based on multiple approaches, including detailed clinical assessment or extended to non-invasive stress studies or coronary angiography. Risk-stratified screening is proposed as an alternative to subjecting most patients to coronary angiography, and non-invasive evaluation may help in this approach in improving the yield from subsequent intervention. Studies describing both invasive and non-invasive approaches showed mixed results. There are potential pitfalls of NISS which need further evaluation. One of the important challenges in extrapolating evidence from previous studies with NISS in such patients is that many have included patients with prior vascular disease and diabetes mellitus. To our knowledge our study is the first of its kind in defining the cohort into distinct groups based on presence of diabetes and/or prior vascular disease.

This study describes the clinical use and impact of NISS over a 6-year period in the UK’s largest renal transplant center. Risk-stratified screening was applied to the vast majority of 918 patients who were waitlisted for kidney transplantation. Among 623 asymptomatic patients without diabetes or cardiovascular disease, risk-stratified screening helped avoid NISS in 41% of patients. This is similar to the 43% of 514 patients, defined as in a ‘low-risk’ category in the study by Kasiske et al. [7]. Despite this stratification, 368 asymptomatic patients in our study were deemed as needing NISS for ‘high-risk factors’ including age, prior RRT, smoking and hyperlipidemia. Although 14% of these patients had abnormal NISS, none of those who subsequently underwent coronary angiography were noted to have occlusive coronary artery disease needing intervention. Patel et al. [10] studied 300 patients including some with prior diabetes and vascular disease; 97 patients underwent coronary angiography, but only 17 of these patients proceeded with interventions. It was difficult to attribute differences in mortality to interventions, and the authors speculated that mortality was more likely to be the consequence of co-morbidity, age and failure to list for kidney transplantation. In our study 178 asymptomatic patients out of 318 (56%) patients with ‘normal NISS’ went on to receive kidney transplants, compared to 27 out of 50 (54%) patients with ‘abnormal NISS’.

There was no significant association of subsequent mortality or MACE with presence or absence of ischemia on NISS. Patients who developed MACE/death during follow-up had classical baseline risk factors of higher co-morbidity, baseline renal replacement therapy and more AHA risk factors. The one factor which had the strongest influence on clinical outcome was successful transplantation after listing. This effect was seen in all the groups in waitlisted patients including those considered to harbor the highest risk.

There is little evidence in the literature regarding the time patients spend during ‘prelisting evaluation’. Our study provides valuable insights into this, although it is difficult to compare this with standards or practices at other centers. However, our study clearly shows that NISS adds time to pre-transplant evaluation and delays listing. Patients in the so called ‘higher-risk group’ (without DM or CVD) had longer ‘time to listing’ and this increased further in patients who had ‘abnormal results’ with NISS. The need to seek a cardiological opinion and thereafter further assessment with coronary angiography added additional time prior to listing. The benefit of this approach when the yield, in terms of intervention, is low (to non-existent) is therefore highly questionable. This is especially so with the increasing drive to achieve pre-emptive transplantation because of the recognized significant improvement in outcomes with this approach. In our study over 75% of patients were seen in the pre-transplant evaluation clinic prior to commencement of dialysis, in comparison to previous studies describing this pre-emptive assessment rate at 45 to 55%. Time spent with pretransplant evaluation would mean that patients may lose the opportunity for timely listing unless these screening studies are brought further forward in the investigative journey of patients with advanced CKD. 106 of the 719 (15%) patients in our study who initiated evaluation in the preemptive phase had progressive renal impairment and commenced dialysis by the time they were listed.

In this study, analyses in 295 patients with diabetes and/or pre-existing cardiovascular disease helped understanding of whether patients should be classified into distinct groups. Very few baseline features were significantly different in these 2 groups, possibly because of the fact that 44% of patients with CVD had pre-existing DM. However, only 8 of the 295 (2.7%) patients in this group proceeded to require coronary interventions prior to listing. Symptoms of cardiac disease may be difficult to differentiate from kidney disease per se and sensitivity / specificity of symptoms for obstructive coronary disease was shown to be low [22]. It is possible that this may be one of the reasons behind the low pick up rate of obstructive coronary disease in this study. ‘Time to list’ rates were similar to those in Group 2 suggesting that NISS may be the common factor contributing to this time. Adverse-event free rates were higher in those with preexisting cardiovascular disease (compared to those with diabetes alone) although numbers did not reach statistical significance. However, as was seen in patients without DM or CVD, mortality and MACE outcomes during follow up were largely influenced by successful transplantation after waitlisting. Using transplantation as a time-dependent variable the HR for mortality/MACE in those receiving kidney transplants was 0.95 (Table 4).

Our study has certain limitations. Most importantly, this was a retrospective analysis of patients prospectively followed up over the last decade. Small sample size in some groups may influence the results. It is plausible that some cardiovascular events may have been missed during follow up. However, given that patients with or without a transplant were followed up at just 2 centres, and the 2 regional cardiac intervention centres were located within the same NHS Trusts, this is unlikely to have confounded the results as such numbers will have been small. A relatively higher proportion of patients underwent pre-emptive evaluation and the prevalence of severe occlusive coronary disease may be different in centres with very long wait-times. Also, the incidence of composite endpoints is low post-transplantation in our study. Although one may speculate that this could be from short follow up period after transplantation, recent studies showed similarly low rates in the first 30-days post transplantation [23]. The comorbid factors in our study are similar to other larger studies reported previously [24–26]. 124 of the 918 (14%) waitlisted patients had repeat non-invasive stress studies whilst on the transplant list. It was not possible to determine the effect of these re-screening studies on overall outcome. Kasiske et al. [7] noted that follow up screening was recorded in 11.9% of 514 patients studied, despite their centre recommending follow up testing in high risk individuals. Studies such as the CARSK [27] and Ischemia-CKD [28] studies will likely help in guiding the screening strategy before and after listing. The recently reported randomised trial [28] failed to show reduction of death or non-fatal MI with initial invasive strategy (compared to initial conservative approach) in patients with advanced CKD and stable, but with moderate to severe ischemia. Rather invasive strategy was associated with higher incidence of stroke and higher incidence of death or initiation of dialysis. However only 13% were on transplant wait list in the study cohort. Lastly severity of reversible ischemia that was considered as threshold for coronary angiography in our patient cohort may have influenced the findings.

Given the retrospective nature of our study, changes in drug therapy during the follow up that may have influenced the outcomes was not captured in analyses. In this study 52 patients underwent a second form of NISS (mostly stress echocardiography) after the first NISS (MPS) showed features of ischemia but only 5 of these had consistent features of ischemia on second study and underwent coronary angiography prior to listing. This may well reflect the limitations with sensitivity and specificity of NISS [29]. This strategy was used on a case-by-case basis and cannot be recommended for wider application. It is plausible that screening has value in evaluating patients who are very high risk or with multiple comorbidities and could help in reassuring clinicians and patients prior to excluding from waitlisting, as suggested by the UK Renal Association Clinical Practice guidance [30]. However, that alone could not be considered as a valid reason for undertaking NISS in low to modest risk individuals.

The main strength of the study is inclusion of all patients including the ones who did not proceed with transplant listing within the whole cohort. There is a relative paucity of literature investigating the reasons behind exclusion, but our rates of exclusion appear similar (9–13%) [31, 32] as did the finding that medical reasons constitute the bulk of decisions for exclusion [33, 34]. However, to our knowledge this is the only study describing the details of NISS in the ‘Unlisted’ group and offering comparisons with those in the waitlisted groups. Despite the inclusion of this ‘Unlisted’ group it is evident from this study that NISS added no benefit to pretransplant evaluation in 666 patients without DM or CVD and helped changed the course of management in only 19 of 387 (5%) patients with DM or CVD. Our study clearly illustrates that NISS contributes to a cascade of additional investigations adding financial costs to the health care systems and adds valuable time to transplant listing pathways. With resources being finite, there is increasing need to develop strategies to help sustain health care costs by reducing waste. Risk stratification to identify patients who would truly benefit from screening tests needs further refinement and would promote the Choosing Wisely [35] approach.

In summary our study shows that screening with Non-Invasive Stress Studies for coronary artery disease during pretransplant evaluation adds no value in patients without diabetes or cardiovascular disease. Our study reiterates the strong benefit of successful transplantation in improving outcomes in patients with differing risk profile. NISS is associated with many costs, including additional time taken in the pretransplant evaluation pathway thereby leading to delay in transplant listing, notwithstanding the financial costs. There is a strong need for further studies to determine the effectiveness of screening studies in pretransplant evaluation. Until then screening based on detailed clinical assessment may be an adequate strategy to assist in timely listing of candidates without diabetes or cardiovascular disease.

## Supporting information

S1 Table Baseline characteristics in waitlisted candidates in Group 2 based on NISS–normal vs abnormal NISS. (DOCX)

S1 Fig (JPG)

S1 Dataset (XLSX)
