# Supplementary material for: Non-invasive cardiac stress studies may not offer significant benefit in pre-kidney transplant evaluation: A retrospective cohort study
Source: PLoS One. 2020 Oct 28;15(10):e0240912. doi: 10.1371/journal.pone.0240912 (PMC7592791; doi:10.1371/journal.pone.0240912)
Supplement: S1 Table — (DOCX) [file pone.0240912.s001.docx]

**S1 Table: Baseline characteristics in Waitlisted Candidates in Group 2 based on NISS – Normal vs Abnormal NISS**

|  | | | **NISS Outcome** | | p |
| --- | --- | --- | --- | --- | --- |
|  |  |  | **Normal (n=318)** | **Abnormal (n=50)** |  |
| Age | | | 53.8 (12.0) | 56.2 (10.3) | 0.17 |
| Age Groups | | <35 | 19 (6.0%) | 1 (2.0%) |  |
|  |  | 35-50 | 98 (30.8%) | 13 (26.0%) |  |
|  |  | 50-65 | 131 (41.2%) | 22 (44.0%) |  |
|  |  | > 65 | 70 (22.0%) | 14 (28.0%) |  |
| BMI | | | 26.8 (5.0) | 27.5 (4.6) | 0.35 |
| Male (%) | | | 153 (48.1%) | 38 (76.0%) | < 0.001 |
| Hypertension (%) | | | 284 (89.3%) | 43 (86.0%) | 0.49 |
| Never Smoked (%) | | | 186 (58.5%) | 24 (48.0%) | 0.30 |
| Ethnicity (%) | Caucasian | | 251 (78.9%) | 42 (84.0%) | 0.41 |
| Primary Disease (%) | Immune Kidney Disease | | 102 (32.1%) | 15 (30.0%) | 0.65 |
|  | Hypertensive CKD | | 51 (16.0%) | 7 (14.0%) |  |
|  | Polycystic Kidney Disease | | 56 (17.6%) | 11 (22.0%) |  |
|  | Other | | 64 (39.3%) | 12 (34.0%) |  |
| Previous Transplant (%) | | | 47 (14.8%) | 9 (18.0%) | 0.56 |
| Pre-emptive (%) | | | 243 (76.4%) | 36 (72.0%) | 0.50 |
| Pulmonary Disease (%) | | | 30 (9.4%) | 4 (8%) | 0.99 |
| Peptic ulcer disease (%) | | | 20 (18.1%) | 1 (2.9%) | 0.33 |
| Liver Disease (%) | | | 10 (3.1%) | 2 (4.0%) | 0.67 |
| Previous Cancer (%) | | | 21 (6.6%) | 6 (12.0%) | 0.17 |
| HIV (%) | | | 1 (0.3%) | 1 (2.0%) | 0.25 |
| Charlson Comorbidity Index | | | 2 (2, 2) | 2 (2,3) | 0.46 |
| AHA risk factors (3 or more) | | | 155 (48.7%) | 32 (64%) | 0.05 |
| RAS inhibitors (%) | | | 179 (56.3%) | 31 (62.0%) | 0.45 |
| Statin (%) | | | 174 (54.7%) | 29 (58.0%) | 0.66 |
| eGFR (ml/min/1.73m^2^) | | | 11.6 (4.6) | 11.8 (4.8) | 0.77 |
| Calcium (mmol/L) | | | 2.3 (0.2) | 2.2 (0.1) | 0.14 |
| Phosphate (mmol/L) | | | 1.4 (1.2, 1.6) | 1.5 (1.3, 1.8) | 0.11 |
| PTH (ng/L) | | | 202 (119, 364) | 184 (118, 300) | 0.64 |
| Albumin (g/L) | | | 39.5 (5.7) | 40.9 (4.8) | 0.10 |
| CRP (mg/L) | | | 5 (2.5, 8.7) | 5.3 (2, 8) | 0.71 |
| Total Cholesterol (mmol/L) | | | 4.6 (4.0, 5.4) | 4.5 (3.9, 5.4) | 0.93 |
| HDL Cholesterol (mmol/L) | | | 1.3 (1.1, 1.7) | 1.2 (1.0, 1.6) | 0.33 |
| Total / HDL Cholesterol (mmol/L) | | | 3.4 (2.8, 4.3) | 3.8 (2.7, 4.6) | 0.32 |

*Results shown as Mean (Standard Deviation), Median (IQR) for continuous variables and Number (Frequency) for categorical variables.*

*p-Value from Two Sample T-Test, Mann-Whitney U Test and Chi-Squared or Fisher’s Exact test as appropriate.*
