# Supplementary figures and images for: Non-invasive cardiac stress studies may not offer significant benefit in pre-kidney transplant evaluation: A retrospective cohort study
Source: PLoS One. 2020 Oct 28;15(10):e0240912. doi: 10.1371/journal.pone.0240912 (PMC7592791; doi:10.1371/journal.pone.0240912)

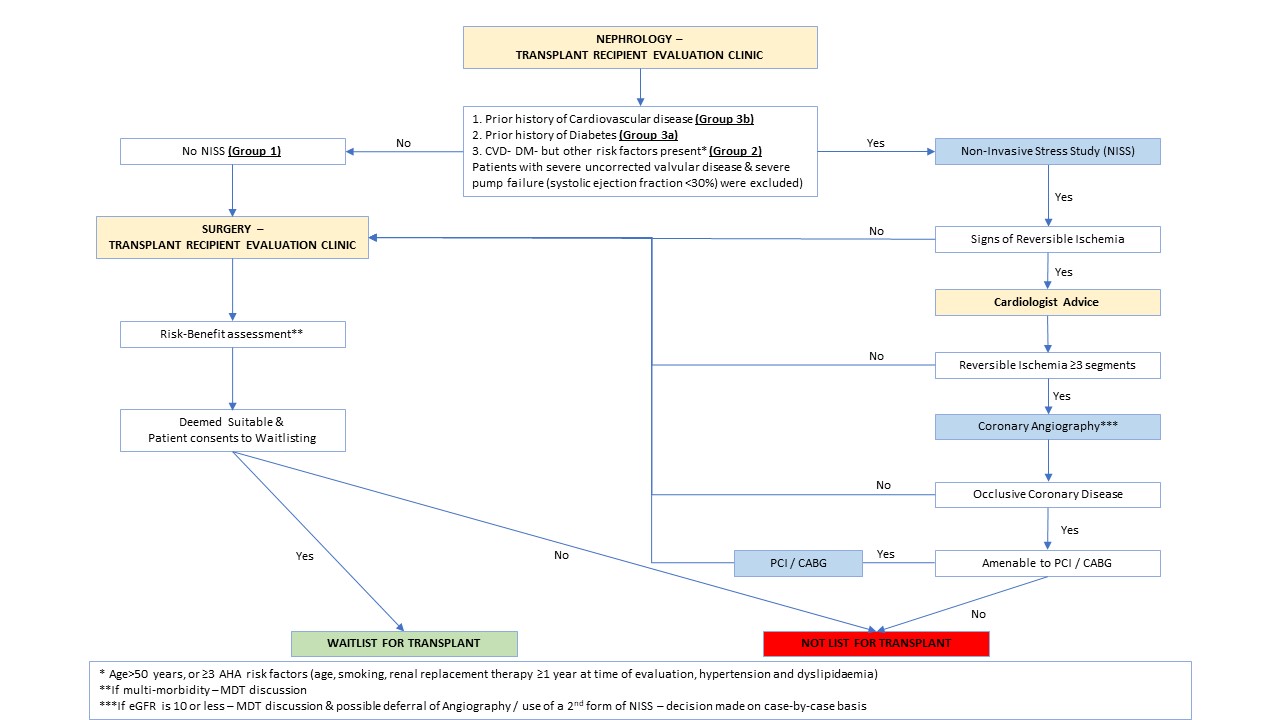

Supplement: S1 Fig — (JPG) [file pone.0240912.s002.JPG]
